# Trade‐off between flight capability and reproduction in Acridoidea (Insecta: Orthoptera)

**DOI:** 10.1002/ece3.8317

**Published:** 2021-11-18

**Authors:** Huihui Chang, Xiaoqiang Guo, Shuli Guo, Nan Yang, Yuan Huang

**Affiliations:** ^1^ College of Life Sciences Shaanxi Normal University Xi’an China; ^2^ Shimen Middle School Foshan China

**Keywords:** Acridoidea, gonadosomatic index, reproduction, trade‐off, wing type

## Abstract

In many insect taxa, there is a well‐established trade‐off between flight capability and reproduction. The wing types of Acridoidea exhibit extremely variability from full length to complete loss in many groups, thus, provide a good model for studying the trade‐off between flight and reproduction. In this study, we completed the sampling of 63 Acridoidea species, measured the body length, wing length, body weight, flight muscle weight, testis and ovary weight, and the relative wing length (RWL), relative flight muscle weight (RFW), and gonadosomatic index (GSI) of different species were statistically analyzed. The results showed that there were significant differences in RWL, RFW, and GSI among Acridoidea species with different wing types. RFW of long‐winged species was significantly higher than that of short‐winged and wingless species (*p* < .01), while GSI of wingless species was higher than that of long‐winged and short‐winged species. The RWL and RFW had a strong positive correlation in species with different wing types (correlation coefficient *r* = .8344 for male and .7269 for female, and *p* < .05), while RFW was strong negatively correlated with GSI (*r* = −.2649 for male and −.5024 for female, and *p* < .05). For Acridoidea species with wing dimorphism, males with relatively long wings had higher RFW than that of females with relatively short wings, while females had higher GSI. Phylogenetic comparative analysis showed that RWL, RFW, and GSI all had phylogenetic signals and phylogenetic dependence. These results revealed that long‐winged individuals are flight capable at the expense of reproduction, while short‐winged and wingless individuals cannot fly, but has greater reproductive output. The results support the trade‐off between flight and reproduction in Acridoidea.

## Graphical abstract

Flight‐capable individuals (long‐winged) of Acridoidea expend energy developing and maintaining the flight apparatus at the expense of reproduction. Flight‐incapable individuals (short‐winged or wingless) cannot fly, but has a greater reproductive output. The results provide evidence for the trade‐off between flight and reproduction in Acridoidea.

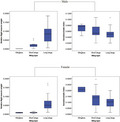

## 1 INTRODUCTION

Birth, growth, reproduction, and death, these components of the life history of an organism are the fundamentals of biological existence. Natural selection operates on life‐history traits, tending to maximize some measure of fitness. This process takes place against a background of trade‐offs and constraints (Dingle, 2006; Roff, 1993; Wootton, 1993). Organisms have finite amounts of resources available for reproduction, growth, metabolism, and maintenance within their whole life history. Increased allocation of resources to any one of these functions necessarily reduces the amount available for the others. Therefore, organisms must reasonably allocate the resources needed for life activities by trade‐off strategies (Conroy et al., 2018; Eizaguirre et al., 2007; Shine, 1997; Tigreros & Davidowitz, 2019; Zhang et al., 2009). Trade‐offs are fundamental to evolutionary biology because they often link the expression of multiple traits, impeding the independent evolution of either trait. Such resource allocation trade‐offs directly affect fitness and underlie the evolution of life histories in all organisms (Guerra, 2011; Gutierrez et al., 2020; Rhainds, 2020; Roff, 1993; Tigreros & Davidowitz, 2019).

Insects occur worldwide and their distributions have been shaped by their ability to fly. The wings of insects vary greatly among different species, and they are more sensitive to the environment. The size and shape of the wings are closely related to the flight and reproduction of insects (Devries et al., 2010; Kunpeng et al., 2020; Menz et al., 2019; Zera, 2016). Flight capability provides an advantage for insects in dispersing, searching for food and mates, but insects utilize a significant portion of their energy budget in building, maintaining, and operating their flight system (Fairbairn & Daphne, 2007; Marden, 2000; Tigreros & Davidowitz, 2019; Zboralski et al., 2015; Zera, 2003; Zhang et al., 2009). The flight muscles of insects represent a major allocation of energy and material. Their flight muscles, for instance, are known to exhibit the highest mass‐specific rates of oxygen consumption of any locomotory tissue (Marden, 2000). Fully grown flight muscles comprise a substantial proportion of total body weight of flight‐capable adults, typically 10%–20% (Zera & Denno, 1997). In order to maintain normal flight capability, the total flight muscle mass of insects must be at least 12%–16% of body mass, and some can even reach 55%–65%. Thus, insects are forced to either make a substantial investment in flight muscle or forego the benefits of aerial travel (Marden, 1989, 2000).

Reproductive investment also is energetically costly and is often limited by available resources (Gutierrez et al., 2020; Papaj, 2000; Steenman et al., 2014). In insects, the energetic and material costs required to fly are likely to divert resources away from other fitness‐related processes. Especially in females, reproductive investment is often limited by the amount of energy reserves that are also used for flight (Elliott & Evenden, 2012; Marden, 2000). Some of the strongest evidence of resource allocation trade‐offs involves the allocation of limited resources between flight muscles and fecundity in wing‐dimorphic insects: a long‐winged morph is flight‐capable at the expense of reproduction, while a short‐winged morph cannot fly, is less mobile, but has greater reproductive output (Conroy et al., 2018; Devries et al., 2010; Elliott & Evenden, 2012; Guerra, 2011; Hayes et al., 2019; Roff, 1986; Tigreros & Davidowitz, 2019; Zera, 2016; Zera & Denno, 1997). This trade‐off occurs as both flight capability and reproduction are energetically costly, and when different traits are each costly, some can be emphasized at the expense of others, and this is an important trade‐off in insects (Guerra & Pollack, 2007; Marden, 2000; Roff, 1986; Zera & Brink, 2000). Similar phenomena of flight–fecundity trade‐offs can be found in many groups, such as bugs (Dingle, 2006), planthoppers (Denno et al., 1989), water striders (Kaitala & Huldn, 1990), crickets (Conroy et al., 2018; Fairbairn & Daphne, 2007; Nanoth Vellichirammal et al., 2014; Zera, 2003, 2016), grasshoppers (Lock et al., 2006; Steenman et al., 2014), aphids (Castaneda et al., 2010; Kobayashi & Ishikawa, 1994), moths (Elliott & Evenden, 2012), parasitoids (Wilson et al., 2020), chrysopids (Khuhro et al., 2014), ants (Azizi et al., 2009; Peeters & Ito, 2001), beetles (Chaudhuri, 2012), and termites (Zhang et al., 2021).

Insects with different types of wing lengths (abbreviated wing type) have different flight capabilities, energy demands, and reproductive abilities (Tigreros & Davidowitz, 2019). As the most diverse order among the polyneopteran insects, Orthoptera has evolved for 300 Mya (Chang et al., 2020; Mugleston et al., 2016; Song et al., 2015). Wing dimorphism is common in Orthoptera insects, and the pattern of flight–fecundity trade‐offs holds can also be found in Orthoptera insects. The comparative studies of crickets and a few other groups of orthoptera insects provide strong support for the trade‐off between flight and reproduction, that is, a highly fecund, short‐winged morph unable to fly and have reduced flight muscles and wings, and a long‐winged morph exhibit developed flight muscles with functional flight apparatus but reduced fecundity (Conroy et al., 2018; Guerra & Pollack, 2007; Lock et al., 2006; Nanoth Vellichirammal et al., 2014; Steenman et al., 2014; Tigreros & Davidowitz, 2019; Zeng & Zhu, 2012; Zera, 2003, 2016; Zera & Brink, 2000; Zhao et al., 2016). Unfortunately, these studies involve only a few species of Orthoptera. Acridoidea (grasshoppers) is a larger family of Orthoptera, with rich diversity, and the forewing and hindwing of its members exhibit variability from full length to complete loss (Cigliano et al., 2021; Song et al., 2015). However, only few studies focused on the trade‐off in Acridoidea insects and have been limited to individual species (Lock et al., 2006; Steenman et al., 2014). In order to verify the trade‐off between flight capability and reproduction at superfamily level, explore the adaptive evolution of wing type, flight muscle, and gonad in Acridoidea insects, we collected and measured the data of body length, wing length, body weight, flight muscle, and gonad traits of grasshoppers, and investigated the differences of wing length, flight muscle, and gonad between species with different wing types, so as to reveal the relationship between flight and reproduction of Acridoidea insects with different wing types.

## 2 MATERIALS AND METHODS

### 2.1 Sample collection and data measurement

A total of 63 species of Acridoidea insects were collected in this study. The information on the samples is shown in Table S1. After collecting samples in the field, we provided some plants from the collection site for the samples to ensure that the samples are alive when the data are measured. The male samples used for analysis were selected as far as possible at the peak of testis development, while the female samples were selected as far as possible at the mature perinatal stage (Han et al., 2018; Hao et al., 2006). Ethyl acetate was used to anesthetize the living grasshopper samples. A vernier caliper with an accuracy of 0.01 mm was used to measure the lengths of body and forewing of male samples of 58 species and female samples of 56 species, respectively. Under the anesthesia of living samples, the flight muscles, ovaries, and testis of sexually mature females and males were taken out, respectively, and weighed with an electronic balance with an accuracy of 0.1 mg. Measurements of all traits were repeated at least three times as long as the number of grasshopper samples allowed, and the final results were averaged. According to the length of wings and the presence or absence of wings, the wing types were divided into three types: long wings (LW) stand for grasshoppers whose forewings surpass two‐thirds of hind femur; short wings (SW) represented grasshoppers whose forewings do not extend two‐thirds of hind femur or have scaly forewings; and wingless (WL) included grasshoppers with completely degraded forewings and hind wings. We selected three typical natural population species of wingless, short wings, and long wings, namely, *Pedopodisma tsinlingensis*, *Haplotropis Brunneriana*, and *Oedaleus Infernalis*, respectively. The body length and wing length of the three species were measured under the three‐dimensional microscopic imaging system, and the anatomical diagrams of their flight muscles, testis, and ovaries were observed. In order to verify the accuracy of the data, we compared and analyzed the measured morphological data by referring to the published data in Biodiversity Heritage Library (BHL, https://www.biodiversitylibrary.org/) (Pilsk et al., 2010) and Orthoptera Species File (OSF, http://orthoptera.speciesfile.org/) (Cigliano et al., 2021), and reviewed the descriptions of related morphological traits of grasshoppers in published literatures and books (Cigliano et al., 2021; Li & Xia, 2006; Xia, 1994; Yin & Xia, 2003; Zheng & Xia, 1998).

### 2.2 Correlation analysis

To better measure differences and correlations in traits between species with different wing types and avoid biased estimation, we use relative wing length (RWL, the ratio of forewing length to body length), relative flight muscle weight (RFW, the ratio of flight muscle weight to body weight), and gonadosomatic index (GSI, the ratio of testis or ovaries weight to body weight) to process the raw data obtained from the measurement, and the three indices were standardized. Then, the differences and correlations of RWL, RFW, and GSI among different species were compared.

Spearman's rank correlation test was used to analyze the correlation among RWL, RFW, and GSI of male adults of 58 species and female adults of 56 species, respectively. In addition, the significance of the differences between traits was compared. All data statistics and calculations were performed in R 3.6.1.

### 2.3 Difference analysis between males and females in wing dimorphism species

In order to facilitate the comparison of wing differences between male and female wings in dimorphic species, we divided the types of wings in more detail, namely, wing types 1–5. Wing type 1 (WT‐1, wingless (WL)): forewings and hind wings completely degenerate. Wing type 2 (WT‐2, scales wings (SW)): wings degenerated into scales, laterally located, usually covering the tympanum, a few do not reach the tympanum. Wing type 3 (WT‐3, short wings (SW)): The forewings are shorter than or just reach two‐thirds of the hind femur and at least adjoin the back. Wing type 4 (WT‐4, relatively long wings (LW)): forewings surpass two‐thirds of hind femur but do not extend the apex. Wing type 5 (WT‐5, long wings (LW)): forewings exceed the apex of hind femur.

Five species with wing dimorphism were selected to analyze the differences in RFW and GSI between male and female individuals with different wing types. These five species all belong to Pamphagidae, namely, *Eotmethis rufemarginis*, *Filchnerella nigritibia*, *F*. *rubimargina*, *F*. *tenggerensis*, and *Pseudotmethis rubimarginis*. The male forewings of the five species belong to WT‐3 (short wings) and the female forewings are WT‐2 (scales wings). First, homogeneity test of variances and normal distribution test were performed on the relevant data. Then, *t*‐test was used to compare the differences of RFW and GSI between male and female individuals of different species. Statistical analysis was completed by R 3.6.1.

### 2.4 Phylogenetic analysis

Complete mitogenome sequences of 39 Acridoidea species and two outgroups of Pyrgomorphoidea (*Mekongiana xiangchengensis* and *Mekongiella kingdoni*) were used to perform phylogenetic analysis. Their accession numbers and information are listed in Table S2. The phylogenetic relationships of 41 species were determined by the dataset of 13 mitochondrial protein‐coding genes (PCGs: *COX1*, *COX2*, *COX3*, *ATP6*, *ATP8*, *CYTB*, *ND1*, *ND2*, *ND3*, *ND4*, *ND4L*, *ND5*, and *ND6*) and two mitochondrial ribosomal RNAs (rRNAs: *rrnL* and *rrnS*) using Bayesian inference (BI). PhyloSuite v1.2.1 (Zhang et al., 2020) was used to extract the required mitochondrial gene sequences. We employed several alignment strategies for different loci in our dataset. For 13 PCGs, after removing the stop codons, we aligned based on the conservation of reading frames by first translating into amino acids in MEGA X (Kumar et al., 2018), aligning individually in MUSCLE (Edgar, 2004) using default parameters, and back‐translating to nucleotides. Two rRNAs were aligned by MAFFT (Katoh et al., 2009) using default parameters. Alignments of individual genes were concatenated by Concatenate Sequence in PhyloSuite v1.2.1 (Zhang et al., 2020). The optimal model for the concatenated matrix was selected in PartitionFinder v.2.1.1 (Lanfear et al., 2012) using a greedy search algorithm and the Bayesian information criterion (BIC). The phylogenetic tree was reconstructed by MrBayes 3.2.6 (Ronquist & Huelsenbeck, 2003) with the MCMC analysis run for 2,000,000 generations and sampling every 1000 trees. After discarding the first 25% samples as burn‐in, posterior probabilities (PP) were calculated in a consensus tree. The resulting trees were visualized using iTOL (Letunic & Bork, 2019).

### 2.5 Phylogenetic signal analysis

If species with close phylogenetic relationship were closer in traits, they would be closer in adaptability, that is, the distribution of traits on the evolutionary tree was not random, but showed certain phylogenetic signals (Cornwell & Nakagawa, 2017). At present, the known phylogenetic relationship can be used to analyze the correlation of traits, which can be measured by different parameters, such as Blomberg's *K* (Blomberg et al., 2003) and Pagel's *λ* (Freckleton et al., 2002; Pagel, 1999). These parameters all describe the strength of the phylogenetic signal, but they are somewhat different from each other (Blomberg et al., 2003; Keck et al., 2016). There are uncertainties in the estimation of phylogenetic signals, such as the uncertainties of phylogenetic trees, traits, and models (Cornwell & Nakagawa, 2017). In order to reduce the impact of these uncertainties on the analysis results as much as possible, judge the phylogenetic signals among different traits more accurately, judge the phylogenetic signals of RWL, RFW, and GSI more accurately, and test the phylogenetic relationships of biological traits among different species, four phylogenetic signal parameters, Pagel's *λ*, Blomberg's *K*, Abouheif's *C*_mean_, and Moran's *I*, were analyzed by ape (Paradis et al., 2004), phytools (Revell, 2012), adephylo (Jombart et al., 2010), picante (Kembel et al., 2010), and other packages in the software R 3.6.1. The Pagel's λ and Blomberg's *K* values were calculated in the Phytools package, and the Abouheif's *C*_mean_ and Moran's *I* values were calculated in the Adephylo package. The Moran's *I* index is the standard measure of autocorrelation used in spatial statistics and has been proposed as a way to measure the phylogenetic signal (Gittleman & Kot, 1990). *I* = 0, traits are similar among species, as predicted under the Brownian motion model. *I* < 0, traits are similar among species, but the similarity is lower than predicted by the model. *I* > 0, closely related species are more similar to traits (Jombart et al., 2010). Abouheif adapted a test for serial independence to detect a phylogenetic signal in phenotypic traits. Abouheif's *C*_mean_ index (*C*) has been shown to be a Moran's *I* index computed with a specific matrix of phylogenetic weights (Pavoine et al., 2008). Pagel's *λ* may be used to characterize the degree of phylogenetic correlation of comparative data and to test data for evidence of phylogenetic dependence. *λ* normally varies between 0 (phylogenetic independence) and 1 (species traits covary in direct proportion to their shared evolutionary history), indicated that the level of phylogenetic dependence is intermediate between the conventional null model of Brownian motion (*λ* = 1) and that of no phylogenetic dependence (*λ* = 0) (Freckleton et al., 2002; Pagel, 1999). Blomberg's *K* is a relatively new phylogenetic signal measurement method. *K* < 1 implies that relatives resemble each other less than expected under Brownian motion evolution along the candidate tree and the similarity of traits among species was less than expected. *K* ≥ 1 implies that close relatives are more similar than expected under Brownian motion evolution and the variation of traits depends on phylogeny and has strong phylogeny signal (Blomberg et al., 2003).

## 3 RESULTS

### 3.1 Statistical analysis of wing types, flight muscles, and gonads

Among the samples of 63 species collected in 11 provinces in China, 58 species have male samples, 56 species have female samples, and 54 species have both female and male samples (Table S3). The results of Kruskal–Wallis tests showed that there were significant differences in RWL, RFW, and GSI among species with different wing types, whether in females or males (*p* < .01).

**FIGURE 1:**
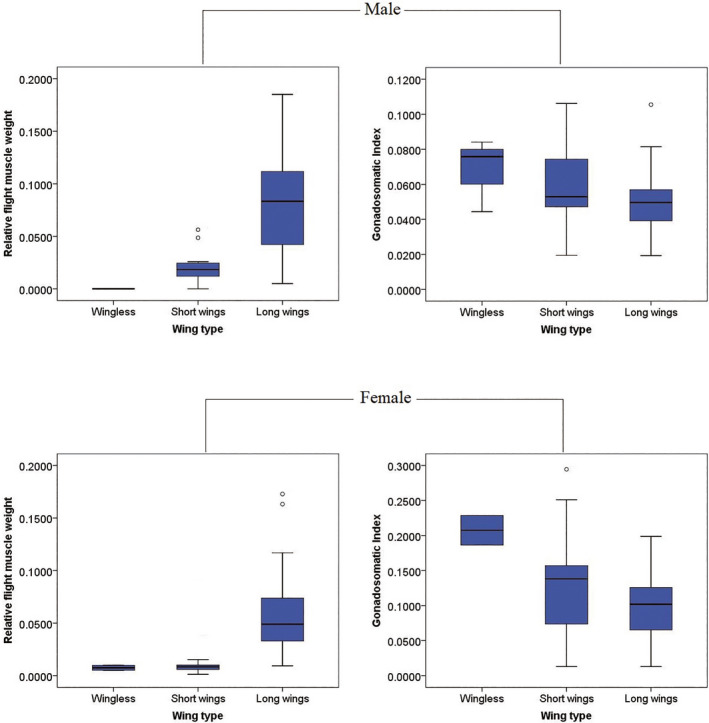
Differences of relative flight muscle weight and gonadosomatic index of Acridoidea species between different wing types

The number of LW, SW, and WL in male species was 39, 16, and 3, respectively (Table S3). The statistical analysis of RFWs and GSIs of males with different wing types are shown in Figure 1. The RFWs of LW male grasshoppers (median: 0.0833, mean: 0.0798) were significantly higher than that of SW species (median: 0.0183, mean: 0.0246) (*p* < .01), and the highest is *Bryodemella holdereri holdereri*, followed by *Sphingonotus yenchihensis*, reaching 0.1851 and 0.1843, respectively. The WL Male grasshoppers have no flight muscle. The GSIs of WL grasshoppers (median: 0.0758, mean: 0.0681) were higher than that of LW (median: 0.0496, mean: 0.0502) and SW (median: 0.0530, mean: 0.0592), and the highest is *Apalacris tonkinensis*, followed by *Pedopodisma emeiensis*, reaching 0.1062 and 0.0901, respectively, while the GSIs of LW were relatively low, *Trilophidia annulata* was only 0.0194, which is 5.47 times worse than the highest GSI.

The number of LW, SW, and WL in female samples was 36, 18, and 2, respectively (Table S3). For female samples, the statistical results of RFWs and GSIs with different wing types could be found in Figure 1. The RFWs of LW females (median: 0.0490, mean: 0.0587) were higher than that of SW (median: 0.0083, mean: 0.0140) and WL (median: 0.0075, mean: 0.0075) species, and the highest was *S*. *ningsianus*, reaching 0.1728. The GSIs of WL grasshoppers (median: 0.2075, mean: 0.2075) were higher than that of LW (median: 0.1019, mean: 0.0984) and SW (median: 0.1382, mean: 0.1364), and the highest was *F*. *tenggerensis* (0.2945), 22.83 times higher than that of the lowest *Mongolotettix japonicus* (0.0129).

**FIGURE 2:**
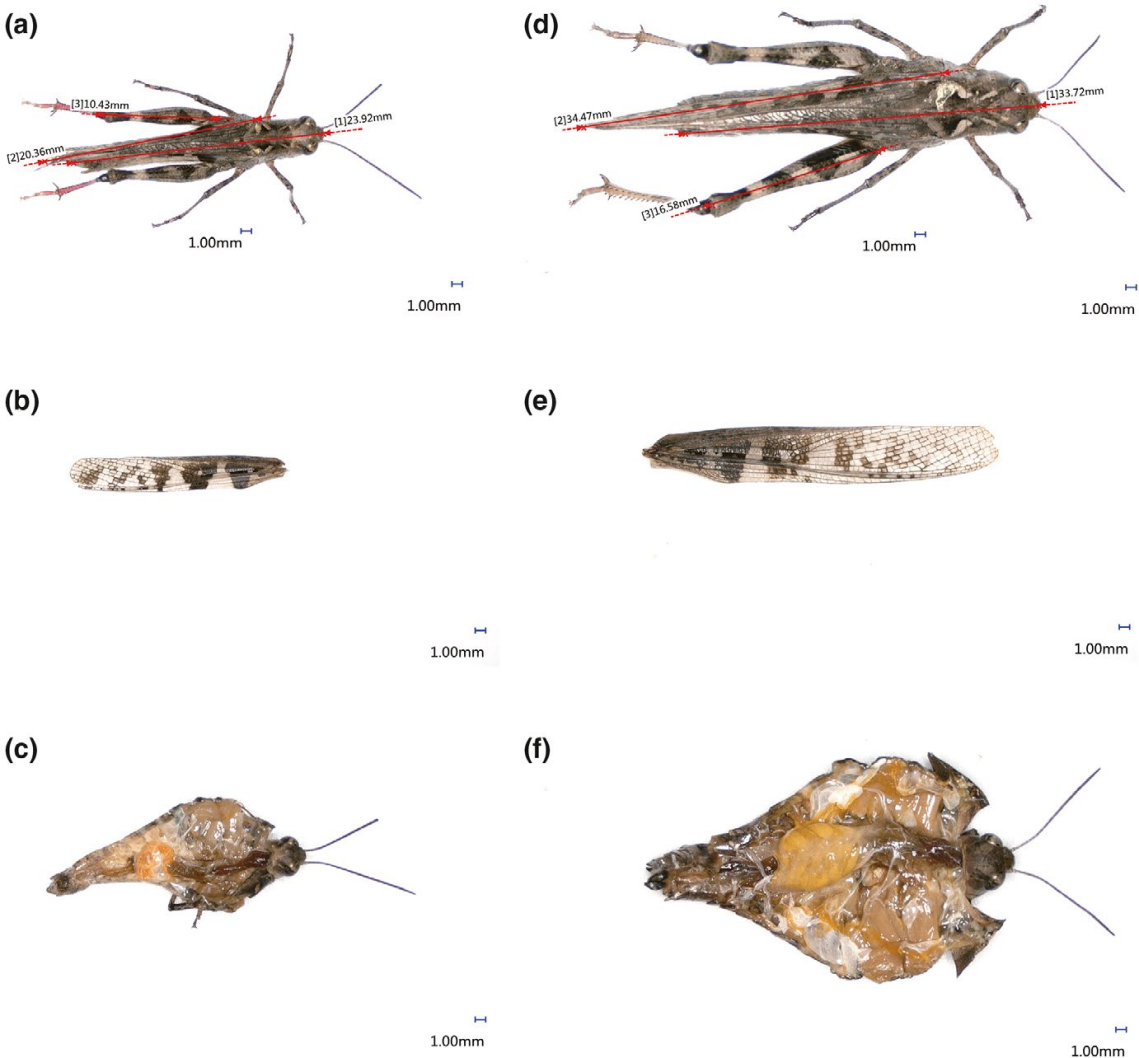
Anatomy images of *Oedaleus infernalis* under three‐dimensional microscopic imaging system (a. Measurement of male body length and wing length; b. Male wing type; c. Anatomy image of male flight muscle and testis; d. Measurement of female body length and wing length; e. Female wing type; f. Anatomy image of female flight muscle and ovary)

**FIGURE 3:**
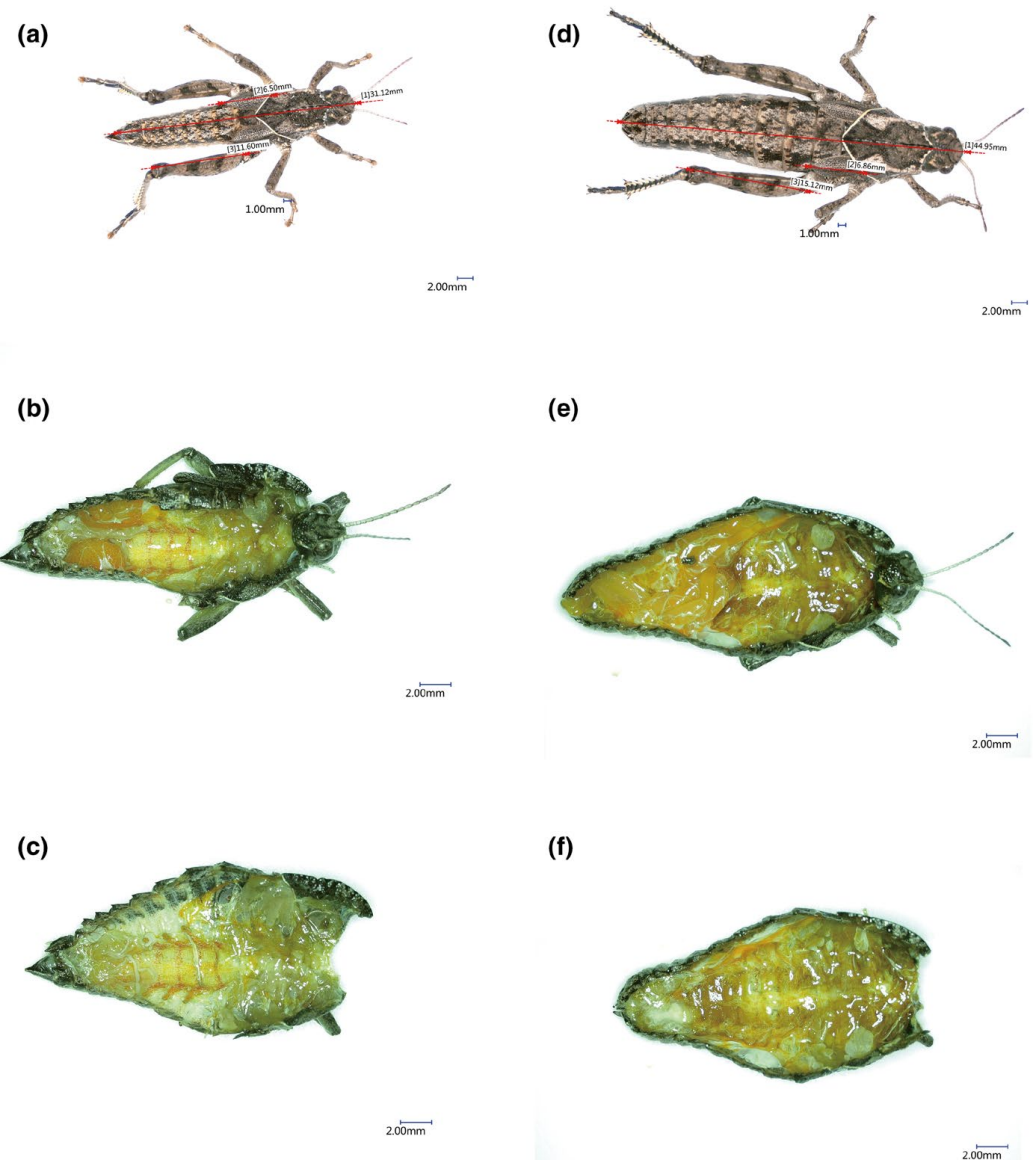
Anatomy images of *Haplotropis brunneriana* under three‐dimensional microscopic imaging system (a. Measurement of male body length and wing length; b. Anatomy image of male testis; c. Anatomy image of male flight muscle; d. Measurement of female body length and wing length; e. Anatomy image of female ovary; f. Anatomy image of female flight muscle)

**FIGURE 4:**
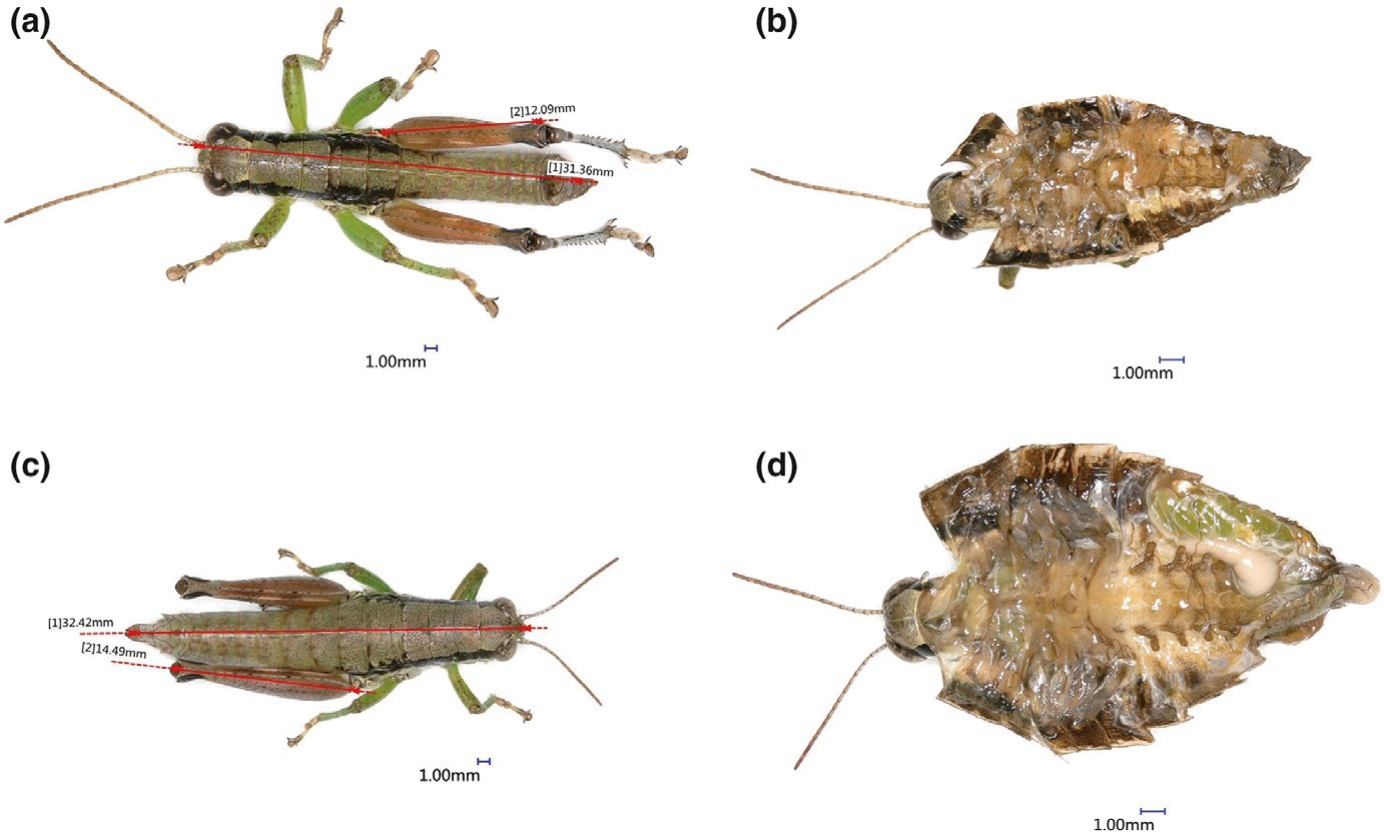
Anatomy images of *Pedopodisma tsinlingensis* under three‐dimensional microscopic imaging system (a. Measurement of male body length; b. Anatomy image of male testis; c. Measurement of female body length; d. Anatomy image of female ovary)

The body length, flight muscle, ovary, and testis of WL, SW, and LW three grasshoppers were observed by three‐dimensional microscopic imaging system. The results showed the same rules: the flight muscle of *Oedaleus infernalis* (LW) was more developed, but the gonad was relatively small (Figure 2), while the gonads of *Haplotropis Brunneriana* (SW) (Figure 3) and *P*. *tsinlingensis* (WL) (Figure 4) were more developed, but the flight muscles were not obvious, *P*. *tsinlingensis* even had no flight muscle.

### 3.2 Correlations among wing length, flight muscle, and gonad

The correlation analysis of RWL, RFW, and GSI of grasshoppers adults with different wing types showed that there was a positive correlation between RWL and RFW in both male and female samples, and the spearman's rank correlation coefficient (*r*) of the two traits in males and females was .8344 and .7269, respectively. There was significant difference in RFW of species with different wing lengths in both males (*p* = 1.379e‐15) and females (*p* = 2.20e‐16) (*p* < .01). The negative correlation between RFW and GSI in both males (*r* = −.2649) and females (*r* = −.5024) could be found. Similarly, the GSI of species with different RFW was significantly different in both males (*p* = .0485) and females (*p* = 7.973e‐5) (*p* < .05).

### 3.3 Differences in flight muscle and gonad between males and females of wing dimorphism species

The comparison results of five species with dimorphism of male and female wings showed that, except for *F*. *rubimargina* (*p* > .05), there were significant differences in RFW of *Eotmethis rufemarginis*, *Pseudotmethis rubimarginis*, *F*. *nigritibia*, and *F*. *tenggerensis* between male and female samples (*p* < .05). The RFW of males (WT‐3, short wings) was higher than that of females (WT‐2, scales wings) in the five species (Table 1). There were significant differences in GSI between male and female samples of the five species (*p* < .05), and the GSIs of females (WT‐2, scales wings) were higher than that of males (WT‐3, short wings) (Table 1).

**TABLE 1:** Comparison of relative flight muscle weight and gonadosomatic index between males and females in wing dimorphism species

Species	Male^a^			Female^b^		
	RFW	GSI	*t* values	RFW	GSI	*t* values
Eotmethis rufemarginis	0.0258 ± 0.0056	0.0538 ± 0.0096	3.6402*	0.0071 ± 0.0004	0.2511 ± 0.0760	−3.5497*
*Filchnerella nigritibia*	0.0208 ± 0.0022	0.0499 ± 0.0023	6.0787*	0.0082 ± 0.0004	0.1259 ± 0.0038	−17.196**
*Filchnerella rubimargina*	0.0181 ± 0.0049	0.0455 ± 0.0062	2.5134	0.0096 ± 0.0015	0.1338 ± 0.0230	−5.997**
*Filchnerella tenggerensis*	0.0132 ± 0.0005	0.0735 ± 0.0026	17.648***	0.0057 ± 0.0003	0.2945 ± 0.0320	−9.7077***
*Pseudotmethis rubimarginis*	0.0233 ± 0.0023	0.0820 ± 0.0107	7.0401**	0.0101 ± 0.0013	0.1329 ± 0.0018	−6.6653**

Abbreviations: GSI, Gonadosomatic index; RFW, relative flight muscle weight.

*p < .05. **p < .01. ***p < .001.

^a^ Wing type of male samples is WT‐2 (wing type 2, scales wings (SW)). The wings degenerated into scales, laterally located, usually covering the tympanum, a few do not reach the tympanum.

^b^ Wing type of female samples is WT‐3 (wing type 3, short wings (SW)). The forewings are shorter than or just reach two‐thirds of the hind femur and at least adjoin the back.

### 3.4 Evolutionary relationship among wing length, flight muscle, and gonad

The phylogenetic relationships of 39 species of Acridoidea are shown in the Figure S1. The tree involved two families: Acrididae and Pamphagidae, which are sister groups. Pamphagidae contains only one subfamily (Thrinchinae). Acrididae consist of two branches, involving nine subfamilies, of which Oedipodinae, Gomphocerinae, and Acridinae form one branch, Calliptaminae, Catantopinae, Eyprepocnemidinae, Hemiacridinae, Melanoplinae, and Oxyinae form another branch.

**FIGURE 5:**
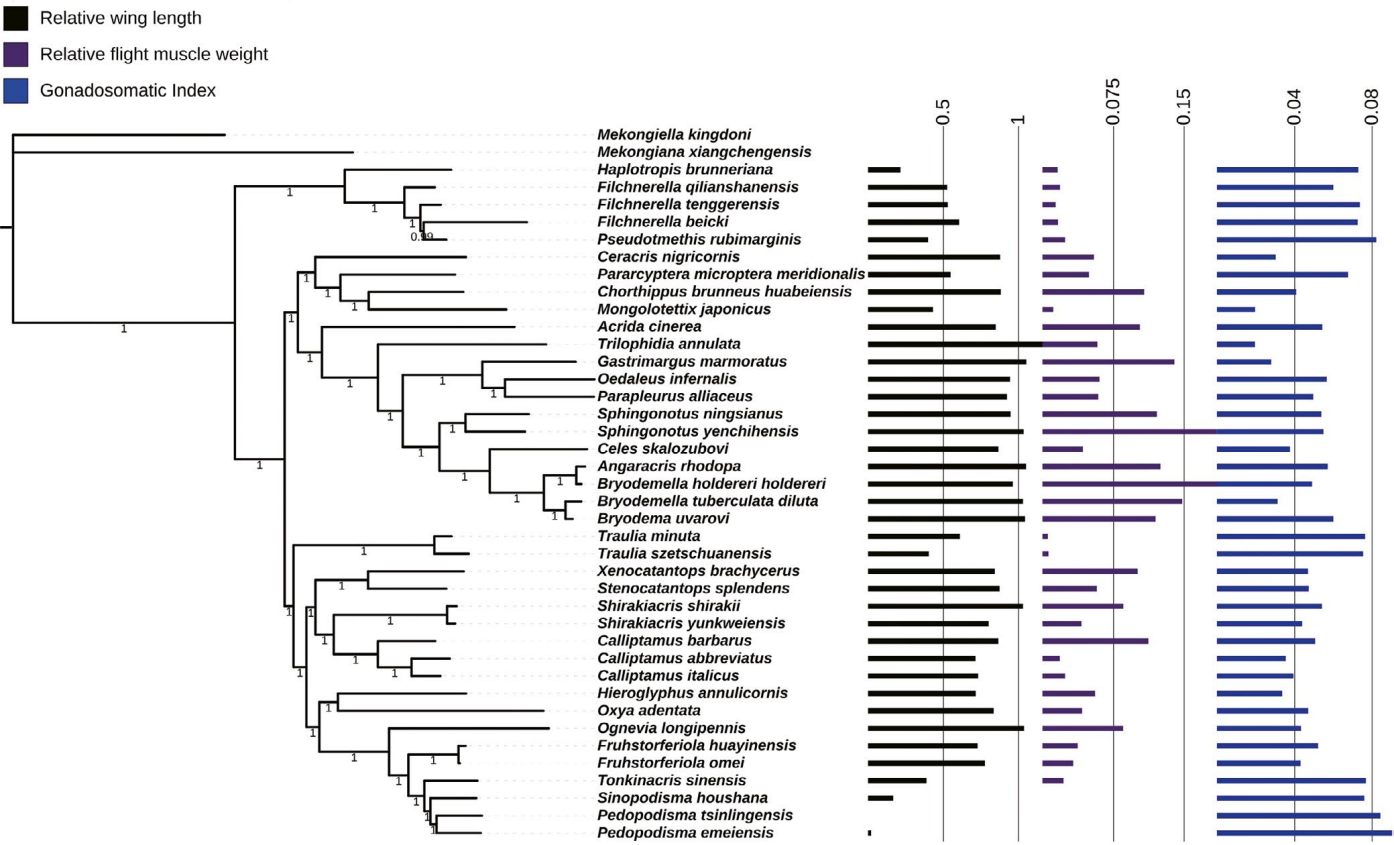
Relative wing length, relative flight muscle weight, and gonadosomatic index of male mapped on the phylogenetic tree of Acridoidea

**FIGURE 6:**
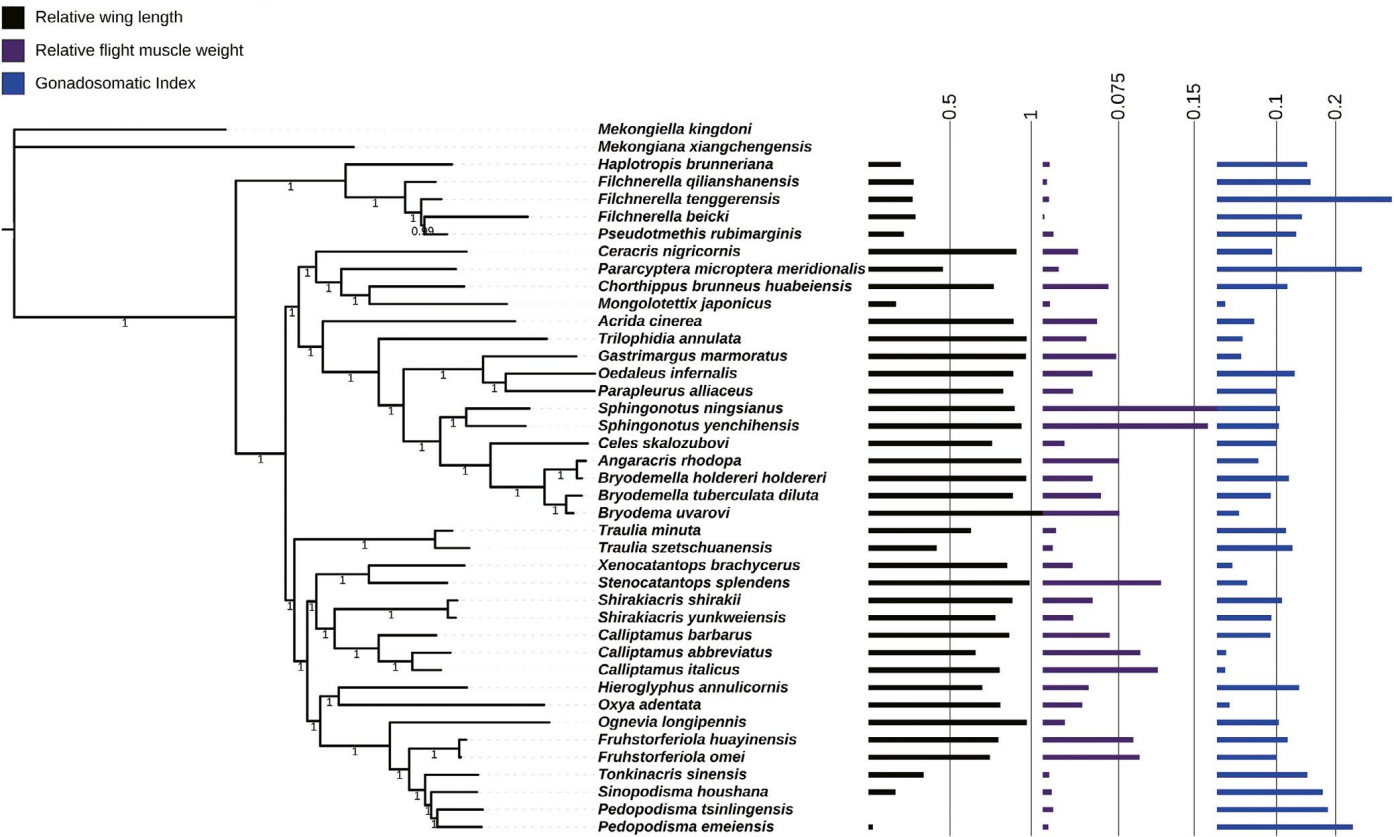
Relative wing length, relative flight muscle weight, and gonadosomatic index of female mapped on the phylogenetic tree of Acridoidea

By fitting RWL, RFW, and GSI of 39 Acridoidea species with phylogenetic tree, it could be found that the greater the RWL, the greater the RFW, and the stronger the flight ability, the smaller the GSI (Figure 5). This rule also existed in females (Figure 6), but the relationship among RWL, RFW, and GSI was more pronounced in females than in males.

### 3.5 Phylogenetic signal analysis of wing length, flight muscle, and gonad

The phylogenetic signal test results from different indices (Pagel's *λ*, Blomberg's *K*, Abouheif's *C*_mean_, and Moran's *I*) showed that RWL, RFW, and GSI of Acridoidea had significant phylogenetic dependence, regardless of female or male (*I* > 0, *C* > 0, *λ* > 0 and *K* > 1, *p* < .05) (Table 2). These traits had particularly significant correlation with phylogeny. The phylogenetic signal of RWL was the strongest, and the four indices were higher than that of RFW and GSI in both males and females. In males, the values of Pagel's *λ* and Blomberg's *K* of RFW were lower than that of GSI, while the values of Abouheif's *C*_mean_ and Moran's *I* were higher than that of GSI, but the differences were not significant. Overall, the phylogenetic signal of RFW was weaker in males. In females, the phylogeny signal of GSI was weaker than that of RWL and RFW (Table 2).

**TABLE 2:** Phylogenetic signals of relative wing length, relative flight muscle weigh, and gonadosomatic index in Acridoidea

Traits	Pagel's *λ*		Blomberg's *K*		Abouheif's *C*_mean_		Moran's *I*	
	Male	Female	Male	Female	Male	Female	Male	Female
RWL	0.9865***	0.9999***	1.6936***	1.7402***	0.6599***	0.6658***	0.6302***	0.6424***
RFW	0.7795***	0.9875***	0.4475**	0.6389**	0.5069***	0.4708***	0.4988***	0.4645**
GSI	0.9113***	0.7286*	0.7092***	0.3612*	0.4805***	0.3438***	0.4566***	0.3327***

Abbreviations: GSI, Gonadosomatic index; RFW, relative flight muscle weight; RWL, relative wing length.

*p < .05. **p < .01. ***p < .001.

## 4 DISCUSSION

Due to the influence of different taxonomic status, living environment, food sources, and other factors, different grasshoppers have different body types, body weights, life forms, and other characteristics (Cigliano et al., 2021; Li & Xia, 2006; Song et al., 2015; Yin & Xia, 2003; Zheng & Xia, 1998). It is of no practical significance and value to directly compare the original data, such as wing length, flight muscle weight, and gonad weight. In order to improve the accuracy and reliability of data, objectively measure the resource investment of grasshoppers in flight and reproduction, better compare the differences in flight capability and reproduction ability of different species, and facilitate the discussion of the differences and correlations between various traits, this study introduced RWL (relative wing length), RFW (relative flight muscle weight), and GSI (gonadosomatic index), respectively, and the indices of the three traits were standardized (Table S3) (Parker et al., 2018).

The comparison of the differences in RFW and GSI of grasshoppers with different wing types showed that the RFW of long‐winged species was significantly higher than that of short‐winged species (*p* < .01), while wingless grasshoppers have no flight muscles. However, the GSIs of short‐winged and wingless species were higher, and the GSI of wingless grasshoppers was higher than that of long‐winged and short‐winged species (Figure 1). These results indicate the trade‐off between flight capability and reproduction in Acridoidea insects among various traits, whether females or males. The long‐winged grasshoppers invested a lot of energy for their flight muscles and short‐winged and wingless species devoted more resources to gonad development. This trade‐off also existed in many insect groups (Azizi et al., 2009; Castaneda et al., 2010; Chaudhuri, 2012; Conroy et al., 2018; Denno et al., 1989; Dingle, 2006; Elliott & Evenden, 2012; Guerra, 2011; Kaitala & Huldn, 1990; Khuhro et al., 2014; Steenman et al., 2014; Tigreros & Davidowitz, 2019; Wilson et al., 2020; Zhang et al., 2021). In addition, the trade‐off between flight capability and reproduction is well known in crickets (Conroy et al., 2018; Guerra & Pollack, 2007; Mole & Zera, 1994; Nanoth Vellichirammal et al., 2014; Zeng & Zhu, 2012; Zera, 2003, 2016; Zera & Brink, 2000; Zhao et al., 2016). Of course, life‐history trade‐offs evidences were also found in other Orthoptera species, such as *Tetrix subulata* (Orthoptera: Tetrigidae) (Lock et al., 2006; Steenman et al., 2014). In this study, it was found that there was a strong positive correlation between RWL and RFW in Acridoidea (*p* < .05). Since wing type and wing length symbolize the flight ability of insects, and grasshoppers with long wings devoted relatively more energy to maintaining their flight ability (Warfvinge et al., 2017; Zeng & Zhu, 2012). Flight is an energy‐consuming and expensive activity and insect flight muscle is the most energetically costly mode of locomotion known: the metabolic rates of flying insects can be 20–100 times that of resting animals (Bartholomew & Casey, 1978; Kammer & Heinrich, 1978; Marden, 2000). In addition to the metabolic cost of flight itself, the cost of maintaining flight muscle and biosynthesis of flight fuel can increase metabolic capacity 4–10 times (Mole & Zera, 1994). Gonadal development in insects is a nutrient‐limited process (Papaj, 2000). The results of this study showed a strong negative correlation between RFW and GSI in Acridoidea (*p* < .05), which further indicated that flight–fecundity trade‐off occurred during the development and growth of grasshoppers. There was a stronger trade‐off between flight and fecundity among capital breeders, so to a certain extent, the degradation or loss of wings led to the weakening of flight ability, which prompted more energy to be invested in reproduction, thereby increasing the GSI and improving reproductive ability (Guerra, 2011; Marden, 2000; Simpson et al., 2011; Stahlschmidt et al., 2020; Tigreros & Davidowitz, 2019).

The comparison of the differences in RFW and GSI between males (WT‐3, short wings) and females (WT‐2, scales wings) with different wing types in five species showed that RFW was proportional to wing length, and GSI was inversely proportional to wing length (Table 1). Males with relatively long wings have higher RFW than females with relatively short wings, while females had higher GSI. Previous studies have shown that females typically invest more into offspring and gamete production than males, who confers advantages in terms of dispersal and finding food and mates (Guerra, 2011; Menz et al., 2019; Zera & Denno, 1997; Zhao et al., 2016). The results of this study also exhibited that females with relatively short wings lack the ability to fly in return for greater reproductive investment (heavier ovaries), while males with relatively long wings may be more prone to dispersal, courtship, and mating (invest in functional flight musculature) at a cost to reproduction (Khuhro et al., 2014; Wilson et al., 2020; Zeng & Zhu, 2012; Zera, 2016). The trade‐off between flight and reproduction was the cause of the differences in flight muscles and gonads of males and females in the species with wing dimorphism, which was also the result of ecological adaptation and evolution (Guerra, 2011; Steenman et al., 2014; Tigreros & Davidowitz, 2019).

According to the results of different traits evolution analysis, the relationship among RWL, RFW, and GSI of Acridoidea insects could be intuitively observed in Figures 5 and 6. In both males and females, the RFW increased with the increase of RWL, while the GSI decreased with the increase of RFW. There was a close correlation between the flight system and the reproductive system of grasshoppers, and this trend (RFW was proportional to RWL, and RFW was inversely proportional to GSI) was even more pronounced in females, which suggested that the flight‐reproduction trade‐off is stronger in females, and is consistent with some previous studies (Conroy et al., 2018; Guerra, 2011; Steenman et al., 2014; Tigreros & Davidowitz, 2019). Females typically invest more into offspring and gamete production than males. By contrast, the cost of reproduction in males can be heavily biased toward competing with rivals for mates, the trade‐off might also be far more evident in traits related to obtaining mates (e.g., courtship and fighting behavior) (Conroy et al., 2018; Guerra, 2011; Steenman et al., 2014).

In this study, the phylogenetic signal analysis of different traits showed that all phylogenetic signal indices (Pagel's *λ*, Blomberg's *K*, Abouheif's *C*_mean_, and Moran's *I*) in both males and females supported the significant phylogenetic dependence of RWL, RFW, and GSI of Acridoidea, among which the phylogenetic signal of RWL was the strongest (Table 2). The phylogenetic signal indices supported that RWL, RFW, and GSI of Acridoidea follow the Brownian motion evolution model, and their variation patterns depend on phylogeny (Blomberg et al., 2003; Pagel, 1999). It can also be found from different branches of phylogenetic tree that species with close relationship, whether male or female, have similar trait indexes and the variation trend among traits is the same. For example, the species of Pamphagidae have relatively high GSI and relatively low RWL and RFW, and the lower the RWL, the lower the RFW, and the higher the GSI. The species of Oedipodinae have relatively low GSI and relatively high RWL and RFW, and the higher the RWL, the higher the RFW, and the lower the GSI. This pattern of change also appears in other branches (Figures 5 and 6). The results of phylogenetic comparative analysis of these traits further verified the previous view, that is, the closer the phylogenetic relationship, the more similar the traits among species, and the closer the adaptability. In other words, the distribution of traits in the evolutionary tree was not random, but showed certain phylogenetic signals (Blomberg et al., 2003; Cornwell & Nakagawa, 2017; Pagel, 1999). In males, the phylogenetic signal of RFW was relatively weak (Blomberg's *K* = 0.4475), which may be due to the fact that flight muscles are an important indicator of flight energy input of grasshoppers, and male grasshoppers showed diversification in flight ability and population communication (Conroy et al., 2018; Steenman et al., 2014; Zhao et al., 2016). In females, the phylogenetic signal of GSI was weaker than that in males. It may be that females are more adaptable to the environment during reproduction than males (Elliott & Evenden, 2012; Guerra, 2011). Therefore, the flight–fecundity trade‐off in Acridoidea is also an adaptive trade‐off formed during long‐term evolution, in which enhancement of one function (e.g., reproduction) has evolved at the expense of another function (e.g., flight ability) (Azizi et al., 2009; Conroy et al., 2018; Steenman et al., 2014; Tigreros & Davidowitz, 2019; Zhang et al., 2021). However, the life‐history trade‐off between flight and reproduction involves multiple aspects (Elliott & Evenden, 2012; Guerra, 2011; Steenman et al., 2014; Tigreros & Davidowitz, 2019). For females, the major penalties of flight capability that are consistent across most taxa are that of a decreased investment into gonads and lowered fecundity (Elliott & Evenden, 2012; Guerra, 2011; Steenman et al., 2014). Indeed, the direction and strength of the trade‐off differ between the sexes and taxa, and with differences in flight capability between the flight morphs (Guerra, 2011). In some cases, the trade‐off exists for males among traits primarily related to mate acquisition and flight‐capable individuals can mitigate the effects of the trade‐off via other traits, for example, by earlier development of flight‐capable individuals relative to flight‐incapable individuals (Guerra & Pollack, 2007; Steenman et al., 2014; Zera & Denno, 1997).

This study used samples from natural populations to explore the evolutionary relationship among wing type, flight muscle, and gonad of the grasshoppers, and provided a basis for future research and the evidence for the trade‐off between flight and reproduction in Acridoidea. However, flight and fecundity in insects were related in a number of ways, for example, physiological constraints (resource‐based trade‐offs), biomechanical constraints (when egg loads affected take‐off performance), adaptive negative correlations (when switching from flight to egg production if appropriate conditions to reproduce were encountered), and adaptive positive correlations (when optimal flight and high fecundity were favored for colonizing new habitats) (Tigreros & Davidowitz, 2019). Therefore, extensive data and research methods are needed to further clarify the effects of different factors on the trade‐offs between flight and reproduction.

## 5 CONCLUSIONS

Based on the measurements of body length, wing length, body weight, flight muscle weight, testis, and ovary weight of 64 Acridoidea species, the differences and correlations of relative wing length (RWL), relative flight muscle weight (RFW), and gonadosomatic index (GSI) of different species were tested in this study. The results showed that there were significant differences in RWL, RFW, and GSI among different species with different wing types or between male and female samples of the species with wing dimorphism. There was a positive correlation between RWL and RFW, while a negative correlation between RFW and GSI. The phylogenetic comparison results also revealed that RWL, RFW, and GSI of Acridoidea species were phylogenetic dependent, which all showed phylogenetic signals. In other words, the results suggested the adaptive evolution of wing length, flight muscle, and gonad, and these traits are related in evolution. Flight‐capable individuals (long‐winged) expend energy developing and maintaining the flight apparatus at the expense of reproduction. Flight‐incapable individuals (short‐winged or wingless) cannot fly but has a greater reproductive output. The results provide evidence for the trade‐off between flight and reproduction in Acridoidea.

## CONFLICT OF INTEREST

The authors declare that they have no competing interests.

## AUTHOR CONTRIBUTIONS

**Huihui Chang:** Conceptualization (supporting); Data curation (equal); Investigation (supporting); Methodology (equal); Visualization (equal); Writing‐original draft (lead); Writing‐review & editing (equal). **Xiaoqiang Guo:** Conceptualization (supporting); Data curation (equal); Investigation (lead); Methodology (equal); Visualization (equal). **Shuli Guo:** Investigation (supporting). **Nan Yang:** Investigation (supporting). **Yuan Huang:** Conceptualization (lead); Funding acquisition (lead); Methodology (equal); Writing‐review & editing (equal).

## Supporting information

Figure S1

Table S1

Table S2

Table S3

## Data Availability

Supplemental files are available at https://datadryad.org/stash/share/QWOtcdDc‐5oU_OzZruXmjL0gG9j4koFtS436YVgxUas.
