# Corrigendum

**DOI:** 10.1002/ece3.10202

**Published:** 2023-06-13

In the article by Chang et al., (2021), there was an error in Table 1. In the table footnote, ‘a’ should represent WT‐3 (wing type 3, short wings (SW)) for males and ‘b’ should represent WT‐2 (wing type 2, scales wings (SW)) for females.

The correct details are as follows:

^a^Wing type of male samples is WT‐3 (wing type 3, short wings (SW)). The forewings are shorter than or just reach two‐thirds of the hind femur and at least adjoin the back.

^b^Wing type of female samples is WT‐2 (wing type 2, scales wings (SW)). The wings degenerated into scales, laterally located, usually covering the tympanum, a few do not reach the tympanum.

The authors would like to apologise for any inconvenience caused.
