# Supplementary figures and images for: Trade‐off between flight capability and reproduction in Acridoidea (Insecta: Orthoptera)
Source: Ecol Evol. 2021 Nov 18;11(23):16849–61. doi: 10.1002/ece3.8317 (PMC8668762; doi:10.1002/ece3.8317)

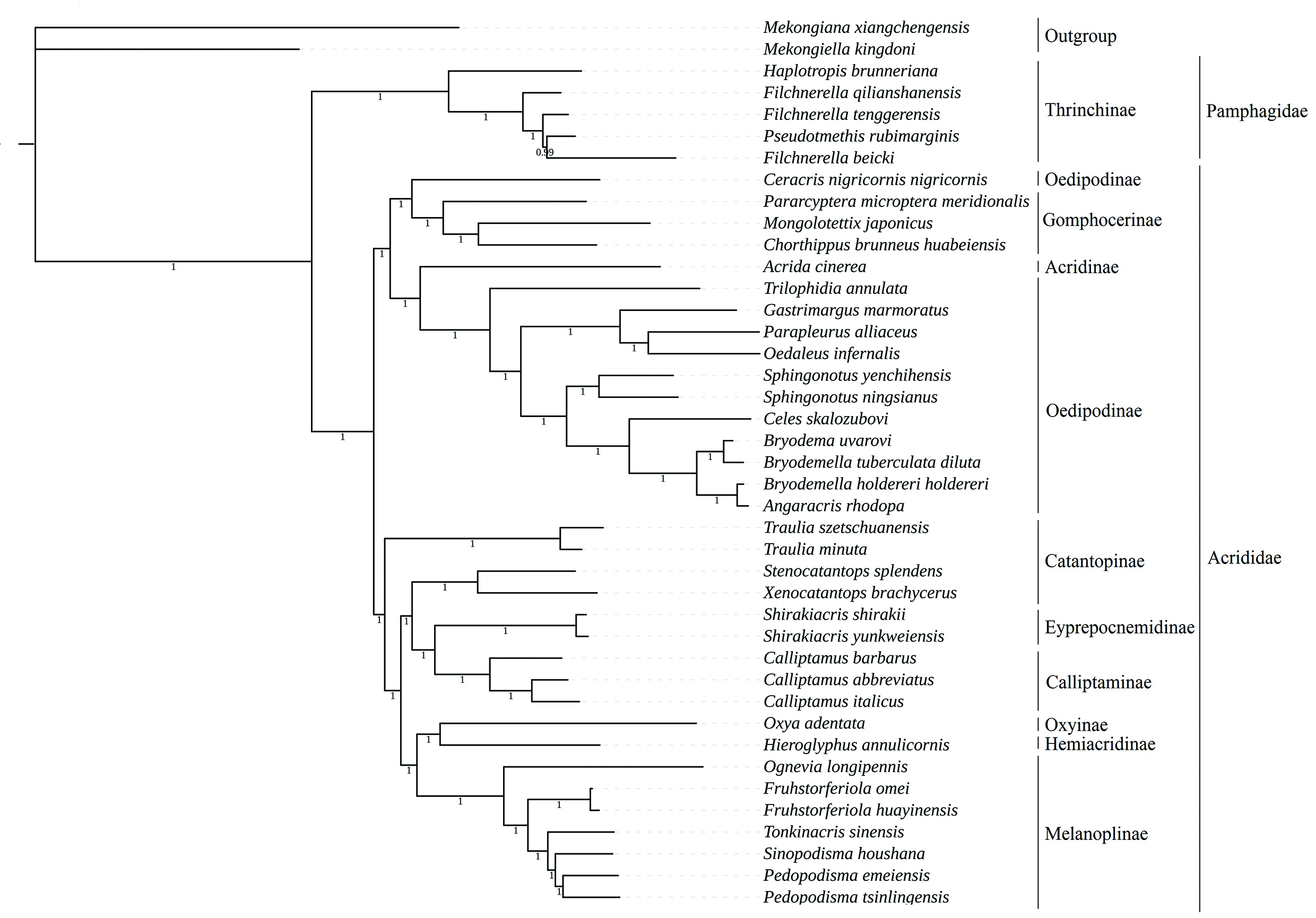

Supplement: Supplementary file 1 — Figure S1 [file ECE3-11-16849-s004.jpg]
