# Supplementary material for: Trade‐off between flight capability and reproduction in Acridoidea (Insecta: Orthoptera)
Source: Ecol Evol. 2021 Nov 18;11(23):16849–61. doi: 10.1002/ece3.8317 (PMC8668762; doi:10.1002/ece3.8317)
Supplement: Supplementary file 2 — Table S1 [file ECE3-11-16849-s002.docx]

**Table S1. Information on the samples used in the present study**

| **Voucher Specimen ID** | **Species** | **Family** | **Gender** | **Collection site** | **Collection date** |
| --- | --- | --- | --- | --- | --- |
| M1830 | *Gastrimargus marmoratus* | Oedipodidae | F/M | Louguantai, Zhouzhi, Shaanxi, China; N108°20'13", E34°3'42" | 2018/7/25 |
| M1831 | *Trilophidia annulata* | Oedipodidae | F/M | Louguantai, Zhouzhi, Shaanxi, China; N108°20'13", E34°3'42" | 2018/7/25 |
| M1832 | *Oedaleus manjius* | Oedipodidae | M | Tianyu River, Zhouzhi, Shaanxi, China; N108°20'13", E34°4'43" | 2018/7/25 |
| 18M02 | *Bryodemella holdereri holdereri* | Oedipodidae | F/M | Gulang, Wuwei, Gansu, China; N37°25'58.73", E102°46'25.51" | 2018/8/8 |
| M1833 | *Bryodema miramae miramae* | Oedipodidae | M | Yitiaoshan Town, Jingtai, Gansu, China; N104°4'45", E37°12'25" | 2018/8/11 |
| 18M07 | *Bryodema nigroptera* | Oedipodidae | M | Yitiaoshan Town, Jingtai, Gansu, China; N104°4'45", E37°12'25" | 2018/8/11 |
| HY-012 | *Angaracris rhodopa* | Oedipodidae | F/M | Gulang, Wuwei, Gansu, China; N37°25'58.73", E102°46'25.51" | 2018/8/8 |
| M1810 | *Sphingonotus ningsianus* | Oedipodidae | F/M | Kangle Grassland, Zhangye, Gansu, China; N38°57'17.67", 99°54'44.31" | 2018/8/6 |
| M1976 | *Oedaleus infernalis* | Oedipodidae | F/M | Shitoukoumen Reservoir, Changchun, Jilin, China; N125°44'11", E43°57'45" | 2019/8/7 |
| M1919 | *Epacromius coerulipes* | Oedipodidae | F/M | Shitoukoumen Reservoir, Changchun, Jilin, China; N125°44'11", E43°57'45" | 2019/8/7 |
| 18M06 | *Celes skalozubovi* | Oedipodidae | F/M | Yan'an, Shaanxi, China; N36°33'4.84", E109°11'39.85" | 2018/8/22 |
| M1811 | *Bryodema dolichoptera* | Oedipodidae | M | Kangle Grassland, Zhangye, Gansu, China; N38°57'17.67", 99°54'44.31" | 2018/8/6 |
| M1805 | *Bryodema uvarovi* | Oedipodidae | F/M | Yitiaoshan Town, Jingtai, Gansu, China; N104°4'45", E37°12'25" | 2018/8/11 |
| M1816 | *Bryodemella tuberculata diluta* | Oedipodidae | F/M | Yan'an, Shaanxi, China; N36°33'4.84", E109°11'39.85" | 2018/8/22 |
| M1807 | *Sphingonotus yenchihensis* | Oedipodidae | F/M | Yitiaoshan Town, Jingtai, Gansu, China; N104°4'45", E37°12'25" | 2018/8/11 |
| 18M03 | *Parapleurus alliaceus* | Oedipodidae | F/M | Songhua Lake, Jilin City, Jilin, China; N126°41'56", E43°43'48" | 2019/8/9 |
| M1834 | *Oxya adentata* | Catantopidae | F/M | Wujiang Town, Zhangye, Gansu, China; N100°26'54", E38°58'59" | 2018/8/6 |
| M1835 | *Oxya agavisa* | Catantopidae | F/M | Emei Mountain, Leshan, Sichuan, China; N29°34'46.85", E103°22'25.20" | 2018/8/27 |
| M1836 | *Fruhstorferiola huayinensis* | Catantopidae | F/M | Ziwuyu, Xi'an, Shaanxi, China; N108°53'39", E34°1'34" | 2018/7/8 |
| M1827 | *Fruhstorferiola omei* | Catantopidae | F/M | Emei Mountain, Leshan, Sichuan, China; N29°34'46.85", E103°22'25.20" | 2018/8/27 |
| M1837 | *Tonkinacris sinensis* | Catantopidae | F/M | Emei Mountain, Leshan, Sichuan, China; N29°34'46.85", E103°22'25.20" | 2018/8/27 |
| HY1701 | *Sinopodisma houshana* | Catantopidae | F/M | Jigong Mountain, Xinyang, Henan, China; N114°4'49", E31°49'56" | 2017/9/11 |
| M1839 | *Pedopodisma tsinlingensis* | Catantopidae | F/M | South Wutai Mountain, Xi'an, Shaanxi, China; N33°59'17.70", E108°58'44.15" | 2018/7/15 |
| 18B09 | *Shirakiacris yunkweiensis* | Catantopidae | F/M | Kangle Grassland, Zhangye, Gansu, China; N38°57'17.67", 99°54'44.31" | 2018/8/6 |
| M1840 | *Xenocatantops brachycerus* | Catantopidae | F/M | Emei Mountain, Leshan, Sichuan, China; N29°34'46.85", E103°22'25.20" | 2018/8/27 |
| M1841 | *Traulia szetshuanensis* | Catantopidae | F/M | Dawuling, Xinyi, Guangdong, China; N111°9'43", E22°15'54" | 2018/8/29 |
| HY1702 | *Traulia minuta* | Catantopidae | F/M | Pu'er, Yunnan, China; N22°49'30.76", E100°57'58.46" | 2017/8/1 |
| M1842 | *Calliptamus italicus* | Catantopidae | F/M | Gulang, Wuwei, Gansu, China; N37°25'58.73", E102°46'25.51" | 2018/8/8 |
| M1843 | *Calliptamus abbreviatus* | Catantopidae | F/M | Louguantai, Zhouzhi, Shaanxi, China; N108°20'13", E34°3'42" | 2018/7/25 |
| M1822 | *Pedopodisma emiensis* | Catantopidae | F/M | Emei Mountain, Leshan, Sichuan, China; N29°34'46.85", E103°22'25.20" | 2018/8/27 |
| M1975 | *Calliptamus barbarus* | Catantopidae | F/M | Suyukou, Helan Mountain, Yinchuan, Ningxia, China; N105°57'15", E38°45'39" | 2019/7/19 |
| M1974 | *Shirakiacris shirakii* | Catantopidae | F/M | Shitoukoumen Reservoir, Changchun, Jilin, China; N125°44'11", E43°57'45" | 2019/8/7 |
| M1909 | *primnoa primnoa* | Catantopidae | F/M | Songhua Lake, Jilin City, Jilin, China; N126°41'56", E43°43'48" | 2019/8/9 |
| M1980 | *Hieroglyphus annulicornis* | Catantopidae | F/M | Mojia Village, Lingchuan, Guilin, Guangxi, China; N110°16'17", E25°14'16" | 2019/9/4 |
| M1817 | *Stenocatantops splendens* | Catantopidae | F/M | Mojia Village, Lingchuan, Guilin, Guangxi, China; N110°16'17", E25°14'16" | 2019/9/4 |
| M1970 | *Apalacris tonkinensis* | Catantopidae | F/M | Sandu Shui Autonomous County, Qiannan, Guizhou, China; N107°53'39", E26°1'19" | 2019/9/2 |
| M1971 | *Sinopodisma rosetllocerca* | Catantopidae | M | Mojia Village, Lingchuan, Guilin, Guangxi, China; N110°16'17", E25°14'16" | 2019/9/4 |
| M1972 | *Oxya intricata* | Catantopidae | M | Mojia Village, Lingchuan, Guilin, Guangxi, China; N110°16'17", E25°14'16" | 2019/9/4 |
| M1973 | *Ognevia longipennis* | Catantopidae | F/M | Songhua Lake, Jilin City, Jilin, China; N126°41'56", E43°43'48" | 2019/8/9 |
| M1904 | *Patanga succincta* | Catantopidae | F | Sandu Shui Autonomous County, Qiannan, Guizhou, China; N107°53'39", E26°1'19" | 2019/9/2 |
| M1913 | *Myrmeleotettix Palpalis* | Gomphoceridae | F | Taibenzhan Town, Ulanhot, Inner Mongolia, China; N122°42'47", E45°44'46" | 2019/8/11 |
| M1968 | *Acrida cinerea* | Acrididae | F/M | Shitoukoumen Reservoir, Changchun, Jilin, China; N125°44'11", E43°57'45" | 2019/8/7 |
| M1845 | *Phlaeoba angustidorsis* | Acrididae | F/M | Emei Mountain, Leshan, Sichuan, China; N29°34'46.85", E103°22'25.20" | 2018/8/27 |
| M1920 | *Mongolotettix vittatus* | Acrididae | F/M | Shitoukoumen Reservoir, Changchun, Jilin, China; N125°44'11", E43°57'45" | 2019/8/7 |
| M1846 | *Mongolotettix japonicus* | Acrididae | F/M | Gulang, Wuwei, Gansu, China; N37°25'58.73", E102°46'25.51" | 2018/8/8 |
| M1847 | *Filchnerella beicki* | Pamphagidae | F/M | Pingshanhu Town, Zhangye, Gansu, China; N100°48'50", E39°4'51" | 2018/8/7 |
| M1848 | *Haplotropis brunneriana* | Pamphagidae | F/M | Miaozuigou Village, Yan'an City, Shaanxi, China; N109°28'43", E36°36'7" | 2018/7/15 |
| M1907 | *Filchnerella rubimargina* | Pamphagidae | F/M | Helan Mountain, Yinchuan, Ningxia, China; N105°57'15", E38°45'39" | 2019/7/19 |
| M1967 | *Pseudotmethis rubimarginis* | Pamphagidae | F/M | Shuimogou, Helan Mountain, Inner Mongolia, China; N105°51'27", E38°58'12" | 2019/7/20 |
| M1908 | *Eotmethis rufemarginis* | Pamphagidae | F/M | Urad Front Banner, Inner Mongolia, China; N108°45'2", E40°47'54" | 2019/7/23 |
| M1905 | *Filchnerella nigritibia* | Pamphagidae | F/M | Helan Mountain, Yinchuan, Ningxia, China; N105°57'15", E38°45'39" | 2019/7/21 |
| M1823 | *Filchnerella tenggerensis* | Pamphagidae | F/M | Yitiaoshan Town, Jingtai, Gansu, China; N104°4'45", E37°12'25" | 2018/8/11 |
| M1808 | *Filchnerella qilianshanensis* | Pamphagidae | F | Gulang, Wuwei, Gansu, China; N37°25'58.73", E102°46'25.51" | 2018/8/8 |
| M1918 | *Tagasta tonkinensis* | Chrotogonidae | F/M | Mojia Village, Lingchuan, Guilin, Guangxi, China; N110°16'17", E25°14'16" | 2019/9/4 |
| M1949 | *Chorthippus albonemus* | Arcypteridae | F/M | Gulang, Wuwei, Gansu, China; N37°25'58.73", E102°46'25.51" | 2018/8/8 |
| M1850 | *Ceracris nigricornis nigricornis* | Arcypteridae | F/M | Louguantai, Zhouzhi, Shaanxi, China; N108°20'13", E34°3'42" | 2018/7/25 |
| M1966 | *Euchorthippus unicolor* | Arcypteridae | F/M | Taibenzhan Town, Ulanhot, Inner Mongolia, China; N122°42'47", E45°44'46" | 2019/8/11 |
| 18W02 | *Chorthippus aethalinus* | Arcypteridae | M | The Moon Lake, Jilin City, Jilin, China; N125°28'36", E43°47'43" | 2019/8/8 |
| 18W04 | *Chorthippus brunneus huabeiensis* | Arcypteridae | F/M | Songhua Lake, Jilin City, Jilin, China; N126°41'56", E43°43'48" | 2019/8/9 |
| M1815 | *Pararcyptera microptera meridionalis* | Arcypteridae | F/M | Gulang, Wuwei, Gansu, China; N37°25'58.73", E102°46'25.51" | 2018/8/8 |
| M0162 | *Atractomorpha lata* | Pyrgomorphidae | F/M | Shitoukoumen Reservoir, Changchun, Jilin, China; N125°44'11", E43°57'45" | 2019/8/7 |
| M1825 | *Atractomorpha psittacina* | Pyrgomorphidae | F/M | Emei Mountain, Leshan, Sichuan, China; N29°34'46.85", E103°22'25.20" | 2018/8/27 |
| M1965 | *Atractomorpha sinensis* | Pyrgomorphidae | F/M | Chang'an, Xi'an, Shaanxi, China; N34°09'13.16", E108°53'50.22" | 2019/10/2 |
| Note: F/M: there are both male and female samples; F: only female samples; M: only male samples. | | | | | |
