# Supplementary material for: Trade‐off between flight capability and reproduction in Acridoidea (Insecta: Orthoptera)
Source: Ecol Evol. 2021 Nov 18;11(23):16849–61. doi: 10.1002/ece3.8317 (PMC8668762; doi:10.1002/ece3.8317)
Supplement: Supplementary file 3 — Table S2 [file ECE3-11-16849-s003.docx]

Table S2. Taxonomic information and GenBank accession numbers for the 41 taxa used in this study

| **Accession number** | **Species** | **Subfamily** | **Family** | **Superfamily** |
| --- | --- | --- | --- | --- |
| NC_014887 | *Acrida cinerea* | Acridinae | Acrididae | Acridoidea |
| NC_025946 | *Angaracris rhodopa* | Oedipodinae | Acrididae | Acridoidea |
| MN083195 | *Bryodema uvarovi* | Oedipodinae | Acrididae | Acridoidea |
| MN083168 | *Bryodemella holdereri holdereri* | Oedipodinae | Acrididae | Acridoidea |
| MN083196 | *Bryodemella tuberculata diluta* | Oedipodinae | Acrididae | Acridoidea |
| NC_030626 | *Calliptamus abbreviatus* | Calliptaminae | Acrididae | Acridoidea |
| MN083183 | *Calliptamus barbarus* | Calliptaminae | Acrididae | Acridoidea |
| NC_011305 | *Calliptamus italicus* | Calliptaminae | Acrididae | Acridoidea |
| MN083197 | *Celes skalozubovi* | Oedipodinae | Acrididae | Acridoidea |
| MK903558 | *Ceracris nigricornis nigricornis* | Oedipodinae | Acrididae | Acridoidea |
| MK903587 | *Chorthippus brunneus huabeiensis* | Gomphocerinae | Acrididae | Acridoidea |
| NC_024923 | *Filchnerella beicki* | Thrinchinae | Pamphagidae | Acridoidea |
| MN083202 | *Filchnerella tenggerensis* | Thrinchinae | Pamphagidae | Acridoidea |
| KU668856 | *Fruhstorferiola huayinensis* | Melanoplinae | Acrididae | Acridoidea |
| MN083184 | *Fruhstorferiola omei* | Melanoplinae | Acrididae | Acridoidea |
| NC_011114 | *Gastrimargus marmoratus* | Oedipodinae | Acrididae | Acridoidea |
| MK903563 | *Haplotropis brunneriana* | Thrinchinae | Pamphagidae | Acridoidea |
| MK903564 | *Hieroglyphus annulicornis* | Hemiacridinae | Acrididae | Acridoidea |
| MK903569 | *Mongolotettix japonicus* | Gomphocerinae | Acrididae | Acridoidea |
| NC_029327 | *Oedaleus infernalis* | Oedipodinae | Acrididae | Acridoidea |
| MK903571 | *Oxya adentata* | Oxyinae | Acrididae | Acridoidea |
| MN083204 | *Pararcyptera microptera meridionalis* | Gomphocerinae | Acrididae | Acridoidea |
| MN083205 | *Pedopodisma emeiensis* | Melanoplinae | Acrididae | Acridoidea |
| KX857635 | *Pedopodisma tsinlingensis* | Melanoplinae | Acrididae | Acridoidea |
| NC_020330 | *Pseudotmethis rubimarginis* | Thrinchinae | Pamphagidae | Acridoidea |
| NC_021610 | *Shirakiacris shirakii* | Eyprepocnemidinae | Acrididae | Acridoidea |
| MN046218 | *Shirakiacris yunkweiensis* | Eyprepocnemidinae | Acrididae | Acridoidea |
| KX857634 | *Sinopodisma houshana* | Melanoplinae | Acrididae | Acridoidea |
| MN083207 | *Sphingonotus ningsianus* | Oedipodinae | Acrididae | Acridoidea |
| MN083191 | *Stenocatantops splendens* | Catantopinae | Acrididae | Acridoidea |
| NC_032716 | *Tonkinacris sinensis* | Melanoplinae | Acrididae | Acridoidea |
| NC_036063 | *Traulia minuta* | Catantopinae | Acrididae | Acridoidea |
| NC_013826 | *Traulia szetschuanensis* | Catantopinae | Acrididae | Acridoidea |
| MK903576 | *Trilophidia annulata* | Oedipodinae | Acrididae | Acridoidea |
| NC_021609 | *Xenocatantops brachycerus* | Catantopinae | Acrididae | Acridoidea |
| MN083208 | *Sphingonotus yenchihensis* | Oedipodinae | Acrididae | Acridoidea |
| MN083172 | *Parapleurus alliaceus* | Oedipodinae | Acrididae | Acridoidea |
| NC_013701 | *Ognevia longipennis* | Melanoplinae | Acrididae | Acridoidea |
| MN083201 | *Filchnerella qilianshanensis* | Thrinchinae | Pamphagidae | Acridoidea |
| NC_014450 | *Mekongiana xiangchengensis* | Pyrgomorphinae | Pyrgomorphidae | Pyrgomorphoidea |
| NC_023921 | *Mekongiella kingdoni* | Pyrgomorphinae | Pyrgomorphidae | Pyrgomorphoidea |
