# Supplementary material for: Trade‐off between flight capability and reproduction in Acridoidea (Insecta: Orthoptera)
Source: Ecol Evol. 2021 Nov 18;11(23):16849–61. doi: 10.1002/ece3.8317 (PMC8668762; doi:10.1002/ece3.8317)
Supplement: Supplementary file 4 — Table S3 [file ECE3-11-16849-s001.docx]

Table S3. The relative wing length, relative flight muscle weight and gonadosomatic index of Acridoidea species

| Species | Number of samples | | Wing types | | Relative wing length | | Relative flight muscle weight | | Gonadosomatic index | |
| --- | --- | --- | --- | --- | --- | --- | --- | --- | --- | --- |
|  | Male | Female | Male | Female | Male | Female | Male | Female | Male | Female |
| *Chorthippus albonemus* | 1 | 1 | WT-4 | WT-4 | 0.79 | 0.688 | 0.1 | 0.052 | 0.1 | 0.12 |
| *Hieroglyphus annulicornis* | 2 | 2 | WT-5 | WT-5 | 0.7133±0.001 | 0.697±0.0063 | 0.0551±0.0016 | 0.0450±0.0023 | 0.0334±0.0014 | 0.1378±0.0011 |
| *Haplotropis brunneriana* | 1 | 1 | WT-2 | WT-2 | 0.21 | 0.196 | 0 | 0.006 | 0.1 | 0.15 |
| *Parapleurus alliaceus* | 1 | 1 | WT-5 | WT-5 | 0.92 | 0.826 | 0.1 | 0.03 | 0 | 0.1 |
| *Celes skalozubovi* | 2 | 2 | WT-5 | WT-5 | 0.8643±0.0063 | 0.7576±0.0017 | 0 | 0.0210±0.0005 | 0.0374±0.0010 | 0.0987±0.0014 |
| *Epacromius coerulipes* | 3 | 3 | WT-5 | WT-5 | 1.0193±0.0097 | 1.0209±0.0009 | 0.0945±0.0135 | 0.0803±0.0051 | 0.0815±0.0079 | 0.1377±0.0038 |
| *Phlaeoba angustidorsis* | 1 | 1 | WT-3 | WT-3 | 0.58 | 0.532 | 0 | 0.01 | 0 | 0.05 |
| *Atractomorpha sinensis* | 2 | 2 | WT-5 | WT-5 | 0.9479±0.0882 | 0.8316±0.0883 | 0.0762±0.1519 | 0.0266±0.0002 | 0.0640±0.0132 | 0.1654±0.0222 |
| *Xenocatantops brachycerus* | 3 | 3 | WT-5 | WT-5 | 0.8402±0.0039 | 0.8501±0.0101 | 0.1003±0.0563 | 0.0291±0.0059 | 0.0467±0.0053 | 0.0250±0.0035 |
| *Calliptamus abbreviatus* | 3 | 2 | WT-3 or WT-4 | WT-3 | 0.7118±0.0084 | 0.6559±0.0298 | 0.0177±0.0008 | 0.0962±0.0091 | 0.0352±0.0059 | 0.0144±0.0026 |
| *Fruhstorferiola omei* | 2 | 2 | WT-5 | WT-5 | 0.7742±0 | 0.7437±0.0026 | 0.0319±0.0003 | 0.0955±0.0160 | 0.0429±0.0033 | 0.1003±0.0014 |
| *Pedopodisma emeiensis* | 1 | 2 | WT-1 | WT-1 | 0 | 0.0232±0.0043 | 0 | 0.0051±0.0006 | 0.1 | 0.2286±0.0387 |
| *Chorthippus aethalinus* | 2 | / | WT-5 | / | 1.0071±0.0019 | / | 0.1275±0.0047 | / | 0.0493±0.0025 | / |
| *Bryodema nigroptera* | 1 | / | WT-5 | / | 1.32 | / | 0.1 | / | 0 | / |
| *Filchnerella nigritibia* | 2 | 3 | WT-3 | WT-2 | 0.2879±0.0024 | 0.1966±0.0019 | 0.0208±0.0022 | 0.0082±0.0004 | 0.0499±0.0023 | 0.1259±0.0038 |
| *Calliptamus barbarus* | 3 | 3 | WT-4 | WT-5 | 0.8638±0.0132 | 0.8628±0.0049 | 0.1117±0.0187 | 0.0659±0.0106 | 0.0504±0.0045 | 0.0891±0.0101 |
| *Angaracris rhodopa* | 3 | 3 | WT-5 | WT-5 | 1.0485±0.0095 | 0.9373±0.0318 | 0.1245±0.0228 | 0.0753±0.0053 | 0.0569±0.0031 | 0.0689±0.0044 |
| *Oedaleus manjius* | 2 | / | WT-5 | / | 0.9238±0.0251 | / | 0.0653±0.0073 | / | 0.0339±0.003 | / |
| *Filchnerella rubimargina* | 3 | 2 | WT-3 | WT-2 | 0.4145±0.0342 | 0.1997±0.011 | 0.0181±0.0049 | 0.0096±0.0015 | 0.0455±0.0062 | 0.1338±0.0446 |
| *Pseudotmethis rubimarginis* | 3 | 3 | WT-3 | WT-2 | 0.3958±0.0074 | 0.2144±0.0105 | 0.0233±0.0023 | 0.0101±0.0013 | 0.0820±0.0107 | 0.1329±0.0018 |
| *Eotmethis rufemarginis* | 3 | 3 | WT-3 | WT-2 | 0.4375±0.0052 | 0.2179±0.0168 | 0.0258±0.0056 | 0.0071±0.0004 | 0.0538±0.0096 | 0.2511±0.0760 |
| *Chorthippus brunneus huabeiensis* | 2 | 2 | WT-5 | WT-5 | 0.8802±0.0068 | 0.7681±0.0027 | 0.1072±0.0007 | 0.0646±0.0012 | 0.0406±0.0025 | 0.1180±0.0446 |
| *Fruhstorferiola huayinensis* | 3 | 3 | WT-4 | WT-4 | 0.7253±0.0024 | 0.7962±0.0323 | 0.0367±0.0017 | 0.0893±0.0162 | 0.0519±0.0049 | 0.1183±0.0101 |
| *Oedaleus infernalis* | 3 | 3 | WT-5 | WT-5 | 0.9416±0.012 | 0.8879±0.0129 | 0.0598±0.0024 | 0.0487±0.0054 | 0.0564±0.0062 | 0.1304±0.0107 |
| *Bryodemella holdereri holdereri* | 2 | 2 | WT-5 | WT-5 | 0.978±0.0192 | 0.9675±0.0017 | 0.1851±0.0402 | 0.0489±0.0009 | 0.0488±0.0022 | 0.1205±0.0026 |
| *Sinopodisma rosetllocerca* | 2 | / | WT-2 | / | 0.1374±0.0012 | / | 0 | / | 0.0488±0.0027 | / |
| *Sinopodisma houshana* | 3 | 3 | WT-1 | WT-2 | 0 | 0.1632±0.004 | 0 | 0.0084±0.0002 | 0.0758±0.0061 | 0.1777±0.0157 |
| *Pararcyptera microptera meridionalis* | 3 | 1 | WT-3 | WT-3 | 0.5458±0.0109 | 0.455 | 0.0486±0.0035 | 0.015 | 0.0674±0.0088 | 0.24 |
| *Myrmeleotettix Palpalis* | / | 1 | / | WT-4 | / | 0.666 | / | 0.033 | / | 0.07 |
| *Atractomorpha psittacina* | / | 3 | / | WT-5 | / | 0.9425±0.0119 | / | 0.0328±0.0050 | / | 0.1501±0.0356 |
| *Bryodemella tuberculata diluta* | 3 | 2 | WT-5 | WT-5 | 1.0279±0.0721 | 0.8856±0.0141 | 0.1476±0.0051 | 0.0572±0.0003 | 0.0310±0.0031 | 0.0898±0.0410 |
| *Sphingonotus ningsianus* | 3 | 2 | WT-5 | WT-5 | 0.9453±0.0108 | 0.8963±0.0019 | 0.1207±0.0309 | 0.1728±0.0029 | 0.0536±0.0043 | 0.1052±0.0009 |
| *Filchnerella beicki* | 1 | 1 | WT-4 | WT-2 | 0.6 | 0.286 | 0 | 0.001 | 0.1 | 0.14 |
| *Filchnerella qilianshanensis* | / | 1 | / | WT-2 | / | 0.274 | / | 0.004 | / | 0.16 |
| *primnoa primnoa* | 2 | 2 | WT-2 | WT-2 | 0.1416±0.0022 | 0.098±0.0131 | 0.0068±0.0009 | 0.0035±0.0003 | 0.0521±0.0026 | 0.1491±0.0003 |
| *Pedopodisma tsinlingensis* | 3 | 2 | WT-1 | WT-1 | 0±0 | 0 | 0 | 0.0098±0.0007 | 0.0841±0.0058 | 0.1864±0.0127 |
| *Bryodema miramae miramae* | 3 | / | WT-5 | / | 1.1487±0.0033 | / | 0.1187±0.0113 | / | 0.0407±0.0009 | / |
| *Ceracris nigricornis nigricornis* | 2 | 2 | WT-5 | WT-5 | 0.8759±0.0593 | 0.9075±0.0196 | 0.0539±0.0084 | 0.0344±0.0017 | 0.0300±0.0037 | 0.0921±0.0154 |
| *Mongolotettix japonicus* | 2 | 2 | WT-3 | WT-3 | 0.4284±0.0022 | 0.1659±0.0009 | 0.0107±0.0009 | 0.0066±0.0013 | 0.0194±0.0124 | 0.0129±0.0001 |
| *Oxya agavisa* | 3 | 3 | WT-4 or WT-5 | WT-4 or WT-5 | 0.7829±0.0384 | 0.6937±0.0556 | 0.0254±0.0015 | 0.0093±0.0008 | 0.0499±0.0037 | 0.1987±0.0170 |
| *Traulia szetshuanensis* | 3 | 3 | WT-3 | WT-3 | 0.4005±0.0308 | 0.4164±0.0043 | 0.0057±0.0003 | 0.0094±0.0011 | 0.0752±0.0021 | 0.1265±0.0080 |
| *Euchorthippus unicolor* | 1 | 1 | WT-5 | WT-5 | 0.81 | 0.825 | 0.1 | 0.062 | 0 | 0.02 |
| *Filchnerella tenggerensis* | 3 | 3 | WT-3 | WT-2 | 0.5269±0.0079 | 0.2685±0.0047 | 0.0132±0.0005 | 0.0057±0.0003 | 0.0735±0.0026 | 0.2945±0.0320 |
| *Mongolotettix vittatus* | 2 | 2 | WT-3 | WT-2 | 0.5196±0.0094 | 0.207±0.0023 | 0.0909±0.0122 | 0.0147±0.0034 | 0.0520±0.0124 | 0.0737±0.0113 |
| *Oxya adentata* | 2 | 2 | WT-4 | WT-4 | 0.8327±0.0036 | 0.808±0.0012 | 0.0413±0.0001 | 0.0387±0.0012 | 0.0468±0.0011 | 0.0202±0.0011 |
| *Oxya intricata* | 1 | 3 | WT-5 | WT-5 | 0.94 | 0.6273±0.0016 | 0 | 0.0126±0.0018 | 0.1 | 0.1157±0.0092 |
| *Traulia minuta* | 3 | / | WT-5 | / | 0.6079±0.0114 | / | 0.0050±0.0006 | / | 0.0762±0.0043 | / |
| *Sphingonotus yenchihensis* | 3 | 2 | WT-5 | WT-5 | 1.025±0.0282 | 0.9384±0.0072 | 0.1843±0.0203 | 0.1632±0.0064 | 0.0547±0.0036 | 0.1035±0.0039 |
| *Calliptamus italicus* | 3 | 2 | WT-5 | WT-4 or WT-5 | 0.7291±0.1117 | 0.8039±0.0841 | 0.0233±0.0018 | 0.1135±0.0101 | 0.0392±0.0046 | 0.0129±0.0008 |
| *Patanga succincta* | / | 1 | / | WT-5 | / | 1.011 | / | 0.04 | / | 0.14 |
| *Bryodema uvarovi* | 1 | 1 | WT-5 | WT-5 | 1.04 | 1.072 | 0.1 | 0.076 | 0.1 | 0.04 |
| *Trilophidia annulata* | 2 | 2 | WT-5 | WT-5 | 1.162±0.0042 | 0.9693±0.0096 | 0.0576±0.0042 | 0.0427±0.0039 | 0.0193±0.0015 | 0.0423±0.0008 |
| *Tagasta tonkinensis* | 3 | 3 | WT-5 | WT-5 | 0.6648±0.0051 | 0.6763±0.0019 | 0.0595±0.0041 | 0.0710±0.0031 | 0.1055±0.0064 | 0.1919±0.0060 |
| *Apalacris tonkinensis* | 2 | 2 | WT-3 | WT-3 | 0.4835±0.0083 | 0.4992±0.0093 | 0.0563±0.0070 | 0.0300±0.0066 | 0.1062±0.0049 | 0.0664±0.0115 |
| *Gastrimargus marmoratus* | 1 | 1 | WT-5 | WT-5 | 1.05 | 0.966 | 0.1 | 0.072 | 0 | 0.04 |
| *Shirakiacris yunkweiensis* | 2 | 2 | WT-5 | WT-5 | 0.7992±0.0216 | 0.778±0.0003 | 0.0107±0.0008 | 0.0297±0.0005 | 0.0437±0.0007 | 0.0909±0.0023 |
| *Bryodema dolichoptera* | 1 | / | WT-5 | / | 1.04 | / | 0.1 | / | 0.1 | / |
| *Shirakiacris shirakii* | 3 | 3 | WT-5 | WT-5 | 1.0279±0.0076 | 0.8829±0.0032 | 0.0851±0.0065 | 0.0490±0.0057 | 0.0539±0.0047 | 0.1089±0.0106 |
| *Ognevia longipennis* | 2 | 1 | WT-5 | WT-5 | 1.0354±0.0177 | 0.971 | 0.0849±0.0046 | 0.021 | 0.0431±0.0054 | 0.1 |
| *Atractomorpha lata* | / | 3 | / | WT-5 | / | 0.8773±0.0091 | / | 0.0419±0.0017 | / | 0.1592±0.0073 |
| *Stenocatantops splendens* | 3 | 2 | WT-5 | WT-5 | 0.8722±0.0041 | 0.9877±0.0025 | 0.0570±0.0024 | 0.1168±0.0006 | 0.0471±0.0029 | 0.0499±0.0041 |
| *Tonkinacris sinensis* | 3 | 3 | WT-3 | WT-3 | 0.3853±0.005 | 0.3362±0.0105 | 0.0216±0.0012 | 0.0059±0.0001 | 0.0766±0.0020 | 0.1518±0.0038 |
| *Acrida cinerea* | 3 | 4 | WT-5 | WT-5 | 0.8463±0.0087 | 0.8898±0.0049 | 0.1027±0.0175 | 0.0533±0.0009 | 0.0541±0.0022 | 0.0618±0.0446 |
| Note: Wing type 1 (WT-1, wingless (WL)): forewings and hind wings completely degenerate. Wing type 2 (WT-2, scales wings (SW)): wings degenerated into scales, laterally located, usually covering the tympanum, a few do not reach the tympanum. Wing type 3 (WT-3, short wings (SW)): The forewings are shorter than or just reaches two-thirds of the hind femur and at least adjoin the back. Wing type 4 (WT-4, relatively long wings (LW)): forewings surpass two-thirds of hind femur but do not extend the apex. Wing type 5 (WT-5, long wings (LW)): forewings exceed the apex of hind femur. | | | | | | | | | | |
